# The effect of geriatric comanagement (GC) in geriatric trauma patients treated in a level 1 trauma setting: A comparison of data before and after the implementation of a certified geriatric trauma center

**DOI:** 10.1371/journal.pone.0244554

**Published:** 2021-01-11

**Authors:** Sascha Halvachizadeh, Lea Gröbli, Till Berk, Kai Oliver Jensen, Christian Hierholzer, Heike A. Bischoff-Ferrari, Roman Pfeifer, Hans-Christoph Pape

**Affiliations:** 1 Department of Trauma, University Hospital Zurich, Zurich, Switzerland; 2 University Zurich, Zurich, Switzerland; 3 Harald Tscherne Research Laboratory, University Hospital Zurich, Zurich, Switzerland; 4 Department of Geriatric Medicine, University Hospital Zurich, Zurich, Switzerland; 5 Centre on Aging and Mobility, University of Zurich, Zurich, Switzerland; 6 Waid City Hospital Zurich, Zurich, Switzerland; Assiut University Faculty of Medicine, EGYPT

## Abstract

**Introduction:**

Improvements in life expectancy imply that an increase of geriatric trauma patients occurs. These patients require special attention due to their multiple comorbidity issues. The aim of this study was to assess the impact of the implementation of geriatric comanagement (GC) on the allocation and clinical outcome of geriatric trauma patients.

**Methods:**

This observational cohort study aims to compare the demographic development and the clinical outcome in geriatric trauma patients (aged 70 years and older) before and after implementation of a certified geriatric trauma center (GC). Geriatric trauma patients admitted between January 1, 2010 and December 31, 2010 were stratified to group pre-GC and admissions between January 1, 2018 and December 31, 2018 to Group post-GC. We excluded patients requiring end-of-life treatment and those who died within 24 h or due to severe traumatic brain injury. Outcome parameters included demographic changes, medical complexity (measured by American Society of Anaesthesiology Score (ASA) and Charlson Comorbidity Index (CCI)), in-hospital mortality and length of hospitalization.

**Results:**

This study includes 626 patients in Group pre-GC (mean age 80.3 ± 6.7 years) and 841 patients in Group post-GC (mean age 81.1 ± 7.3 years). Group pre-GC included 244 (39.0%) males, group post-GC included 361 (42.9%) males. The mean CCI was 4.7 (± 1.8) points in pre-GC and 5.1 (± 2.0) points in post-GC (p <0.001). In Group pre-GC, 100 patients (16.0%) were stratified as ASA 1 compared with 47 patients (5.6%) in Group post-GC (p <0.001). Group pre-GC had significantly less patients stratified as ASA 3 or higher (n = 235, 37.5%) compared with Group post-GC (n = 389, 46.3%, p <0.001). Length of stay (LOS) decreased significantly from 10.4 (± 20.3) days in Group pre-GC to 7.9 (±22.9) days in Group post-GC (p = 0.011). The 30-day mortality rate was comparable amongst these groups (pre-GC 8.8% vs. post-GC 8.9%).

**Conclusion:**

This study appears to support the implementation of a geriatric trauma center, as certain improvements in the patient care were found: Despite a higher CCI and a higher number of patients with higher ASA classifications, Hospital LOS, complication rates and mortality did were not increased after implementation of the CG. The increase in the case numbers supports the fact that a higher degree of specialization leads to a response by admitting physicians, as it exceeded the expectable trend of demographic ageing. We feel that a larger data base, hopefully in a multi center set up should be undertaken to verify these results.

## Introduction

Worldwide, the proportion of adults over the age of 65 years is increasing. In Western societies, more than 25% of trauma admissions are 65 years and older [1], possibly based on a more active lifestyle with an increased risk of injury [2]. In the United States, this increase was calculated to rise to 23.4% by 2060 (compared with 15.2% in 2016) [3], while in Europe, there is an expected to increase of up to 47.5% by 2060 (compared with 2018) [4].

Geriatric trauma patients are expected to present with more comorbidities and higher mortality and morbidity rates [5–7]. Elderly patients present with worse injuries, require longer hospitalisation and make greater use of resources after discharge [8–10]. Concomitant medication have been shown to act as an independent risk factor for the severity of injury [11]. Additionally, elderly have a greater risk of in-hospital complications [12] and their mortality rate is threefold higher when compared with younger patients [13, 14].

Along with these changes in demographics, Geriatric Trauma Centres have been developed [15–17] and certified for ortho-geriatric comanagement (GC). Most central European certification systems propose that patients above the age of 70 years are seen by a geriatrician within 24 hours after hospital admission [18]. According to previous studies, the development of Geriatric Trauma Centres showed beneficial effects on outcome after hip fractures [19]. Moreover, this was also shown for perioperative care by multidisciplinary teams [20]. The focused care appears to improve outcomes even in patients requiring intensive care [20, 21].

Our group has also described, that CG may be useful due to standardization, as achieved by standard operating protocols [18], mobility protocols, and development of special protocols for those with comorbidities and multiple injury scenarios [11, 22, 23].

The aim of this study was to assess the impact of GC on geriatric trauma patients. We hypothesised that patient numbers and the medical complexity of geriatric trauma patients would increase. Further, we hypothesised that the implementation of GC would improve outcomes in geriatric trauma patients.

## Methods

### Study design, ethical consideration, and setting

This study was designed as an observational cohort study and adheres to the STROBE Statement [24]. The local Institutional Review Board and the Ethical Commission “Kantonale Ethikkommission Zurich” approved the study protocol for this study (KEK #2019–01957).

The setting is a level 1 trauma center and bases on an analysis of electronic patients medical records (EMR). These data were extracted automatically by the in-hospital IT-service. Missing and incomplete data and were assessed manually by reviewing the entire patient chart.

### Trauma admission criteria and pre-hospital transported criteria

Trauma admission criteria for our academic Level 1 trauma centre were developed in 2002. They include medical problems, social difficulties and other, non-specified reasons. Medical problems include injuries that either requires surgical treatment or professional medical observation (e.g., mild traumatic brain injuries). Social difficulties include the situation of patients that do not allow outpatient treatment, based on various reasons. The most common reason for trauma admission based on social criteria in the geriatric patient is the lack of support at home. Other reasons for trauma admission include, e.g., regionalisation of patients that live near our hospital and have been treated in other countries.

### Development and implementation of a geriatric comanagement (GC)

The GC was implemented over a period of 2 years, i.e. between 2013 and 2015. This included posting a new academic position for a Professor of Geriatrics, recruiting of specialized nursing staff, rebuilding a clinical ward to allow for in house rehabilitation, implementation of SOP`s, and common ward round strategies [25–27].

### Study population and study size

Geriatric trauma patients, who were hospitalized prior and after the implementation of GC were eligible for this study. In order to avoid a “transition-effect” (including preparatory adjustments, coordination, and fine-tuning after implementation) we included geriatric trauma patients who were treated between January 1^st^ 2010 and December 31^st^ 2010 (stratified to Group pre-GC) or between January 1^st^ 2018 and December 31^st^ 2018 (stratified to Group post-GC). The age-limit for geriatric patients was set at 70 years and above, as foreseen by the criteria for certification as a geriatric trauma center. Patients were followed up until discharge. Patients who died within 24 hours after severe traumatic brain injury, patients with signed “Do Not Resuscitate” (DNR) forms, and patients who deceased from other conditions (e.g. late stage cancer) were excluded from this study.

The study size bases on maximum available data of patients meeting the inclusion criteria. A-priori sample size calculation was not performed.

### Age limit for geriatric assessment

GC based on current recommendations from official certified Geriatric Trauma Centres and includes the collaboration of trauma surgeons with geriatricians for patients aged 70 years and older [28].

### Variables and data sources

Geriatricians are involved in the initial assessment, appropriate adoption of medication and intensive physical therapy. The comparison of admission and discharge notes revealed new pathologic results that were diagnosed during hospitalization. In order to increase comparability, these diagnoses were grouped according to the ICD-10 classification into cardiovascular diagnoses, pulmonary diagnoses, gastrointestinal diagnosis, malnutrition and psychiatric diagnoses. These diagnoses were independent of the trauma-related diagnosis. We only included diagnoses that required further medical attention after discharge.

Medical complexity was based on information at admission as documented. Medical complexity was quantified using the CCI [29] ranging from 0 points (98% estimated 10-year survival) to maximum 37 points (0% estimated 10-year survival) and the pre-operative physical status was quantified by ASA [30] ranging from 1 (healthy person) to 5 (moribund person). The in-hospital course of the present study population includes LOS (days of hospitalization at the department of trauma), and a subgroup analysis for patients that had surgery, and duration from surgery to discharge (days). These data were extracted automatically from the EMR.

Disposition to discharge compared the living facilities or requirements prior to admission with those after discharge. “New nursing home” indicated that the patients did not live in a nursing home prior to admission but required a nursing home after discharge.

Disposition after discharge included the following:

- Return to home,
- Return to the same guided residence (e.g. same nursing home, or retirement home),
- Discharge to rehabilitation unit, new nursing home or
- Transfer to retirement home.

All data were collected based on information by the discharge letter and the EMR. The discharge process in all patients is similar; as soon as the patient is admitted to the hospital, a specialised team plans the discharge. This planning includes estimations of time of discharge, planning the stay after discharge (i.e., rehabilitation centre, nursing home, back to home), and the appropriate application and registrations at both the facilities and the insurance.

These variables were post-hoc further stratified following the main region of injury. These include head injuries, truncal injuries (thoracic and abdominal) and extremity (including pelvic injuries).

### Bias

The inclusion of patients four years prior and after the implementation of GC aimed to increase comparability of routine clinical treatment. In order to avoid selection bias, we aimed to include all geriatric patients that were submitted to conventional treatment and therefore excluded patients with signed DNR or had end of life management. In order to minimize information bias we screened the data for missing and incomplete data and completed them manually by screening the EMR of each patient.

### Statistical methods and quantitative variables

The Shapiro–Wilk test was performed to test for normal distribution. Quantitative variables were summarised as means and standard deviation (±SD) and categorical variables as numbers and percentages. Group comparison on normal distributed quantitative variables was performed using Student’s *t*-test and Pearson’s chi-squared test for categorical variables. Fisher’s exact test was used in the analysis of contingency tables for categorical variables to examine the association between two kinds of treatment strategies (GC vs. non-GC). Missing data were completed by manually screening the EMR. Comparison measures include a 95% confidence interval (CI). Statistically significant difference was assumed at an alpha value of <0.05 (*p* <0.05). All statistical analyses were performed using R (R Core Team, 2018): R is a language and environment for statistical computing (R Foundation for Statistical Computing, Vienna, Austria; URL https://www.R-project.org/).

## Results

### Participants and descriptive data

**Fig 1:**
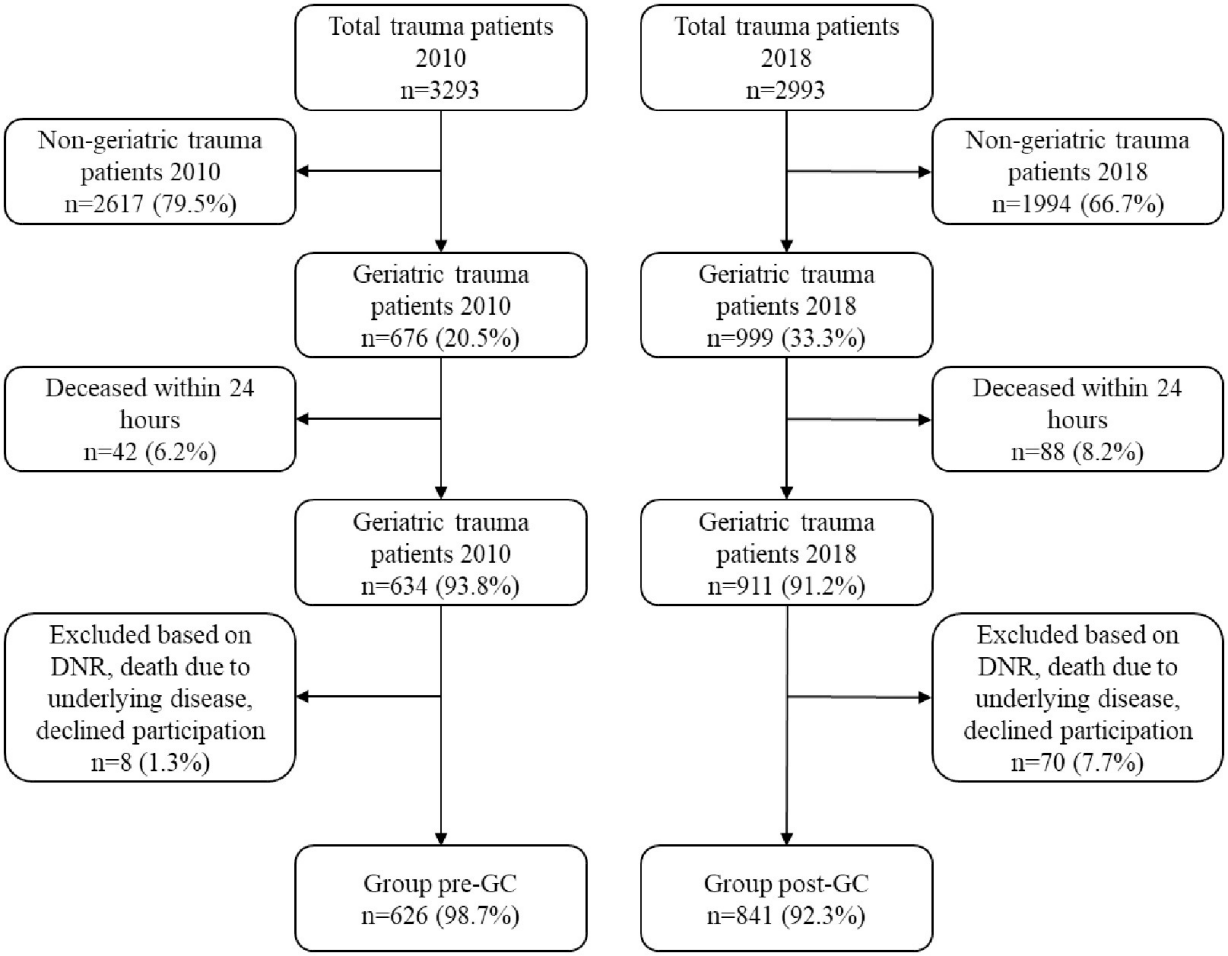
Flow chart of included patients.

Out of total 6286 eligible patients, this study included 1467 geriatric trauma patients (58.8% females, 41.2% males) with a mean age of 80.7 ± 7.0 years, as depicted in Fig 1. Trauma admissions increased by 34.3% when comparing pre-GC (*n* = 626, 42.7%) with post-GC (*n* = 841, 57.3%). The mean age for the post-GC group (81.1 ± 7.3 years) was significantly higher compared with the pre-GC group (80.3 ± 6.7 years) (95% CI = 0.13–1.56, *p* = 0.0206). Descriptive data of study population summarised in Table 1. Most patients suffered from injuries to the head (pre-GC 33.1% versus post-GC 41.3%), followed by injuries to the hip or the femur (8.9% vs. 12.0%), elbow or forearm (7.7% vs 5.2%), abdomen, pelvis, or lumbar spine (5.4% vs. 8.9%), shoulder or humerus (5.1% vs 6.7%), and thorax (6.7% vs. 4.0%).

**Table 1:** Descriptive characteristics of the study population.

	pre-GC	post-GC	*p-value*
*n*	626	841	
Age [years], mean (SD)	80.3 (6.7)	81.1 (7.3)	0.021#
Gender [male], *n* (%)	244 (39.0)	361 (42.9)	n.s+
BMI [kg/m2], mean (SD)	24.8 (3.9)	24.9 (4.6)	n.s.#
Number of surgeries, mean (SD)	1.3 (0.7)	1.2 (0.8)	n.s.#
Non-surgical treatment, n (%)	366 (41.5%)	567 (67.4%)	0.002+
Injury Severity Score, mean (SD)	5.7 (5.3)	6.5 (5.0)	n.s.#

GC = geriatric comanagement

*n* = number

SD = standard deviation

BMI = body mass index

n.s. = not significant

^#^ = Students t-test

^+^ = Pearson chi-square test, Fisher exact test

### In-hospital course

The number of surgeries was comparable in both groups (pre-GC 1.3 ± 0.7 vs. post-GC 1.2 ± 0.8) but the proportion of patients with no surgery was significantly higher in pre-GC (41.5%) when compared with post-GC (67.4%, *p* = 0.002) (Table 1). The LOS decreased significantly post-GC (7.9 ± 14.5 days) when compared pre-GC (10.4 ± 20.3 days) (95% CI = 0.59–4.42, *p* = 0.0102). Times from admission to first surgery and from last surgery to discharge were comparable in both groups (Table 2).

**Table 2:** In hospital course before and after development of a GC.

	pre-GC	post-GC	*p-value*
*n*	626	841	
LOS [day], mean (SD)	10.4 (20.3)	7.9 (14.5)	0.011#
Days from admission to surgery, mean (SD)	3.7 (5.9)	3.9 (6.5)	n.s.#
Days from first surgery to discharge, mean (SD)	8.8 (10.4)	7.9 (22.9)	n.s.#
Duration surgery [minutes], mean (SD)	89.7 (76.3)	100.3 (78.6)	n.s.#

GC = geriatric comanagement

*n* = number

SD = standard deviation

LOS = length of stay

n.s. = not significant

^#^ = Student’s t-test

### New diagnoses and mortality

During hospitalisation, in pre-GC group experienced 1886 new pathological conditions were diagnosed (mean of 3 new diagnoses per patient). In the post-GC group, 7934 new pathological conditions were diagnosed (mean of 9.4 new diagnoses per patient) (Table 3). The rate of diagnosed malnutrition increased to 1.4%. Electrolyte imbalance was diagnosed more frequently in the post-GC group (1.0% vs. 0.3%, *p* = 0.004).

**Table 3:** New findings and diagnoses after hospital admission.

	pre-GC	post-GC	*p-value*
*n*	1886	7934	
Cardiovascular, *n* (%)	203 (10.7)	930 (11.7)	n.s.+
Pulmonary, *n* (%)	19 (1.0)	97 (1.2)	n.s.+
Gastrointestinal, *n* (%)	24 (1.3)	116 (1.5)	n.s.+
Malnutrition, *n* (%)	0 (0.0)	115 (1.4)	NA
Psychiatric, *n* (%)	42 (2.2)	217 (2.7)	n.s.+

GC = geriatric comanagement

*n* = number

n.s. = not significant

^+^ = Pearson chi-square test, Fisher exact test

### Medical complexity

On admission, the post-GC group had a significantly higher admission CCI (5.1 ± 2.0 points vs. 4.7 ± 1.8 points, 95% CI = 0.17–0.57, *p* < 0.001) and a significantly higher age-adjusted admission CCI (1.2 ± 1.7 points vs. 1.6 ± 1.9 points, *p* < 0.001). Patients in the post-GC group were significantly less often classified as ASA 1 or 2 (53.7%) compared with the pre-GC group (62.3.0%, *p* < 0.001) but were significantly more often classified as ASA 3 or higher (46.2%) compared with the pre-GC group (37.5%, *p* < 0.001). Despite the increased medical complexity in Group post-GC, the mortality rate remained comparable (8.3% post-GC vs. 8.8% pre-GC). (Table 4).

**Table 4:** Admission medical complexity (CCI and ASA score) and 30 day mortality rate.

	pre-GC	post-GC	*p-value*
*n*	626	841	
CCI [points], mean (SD)	4.7 (1.8)	5.1 (2.0)	<0.001#
CCI adjusted for age [points], mean (SD)	1.2 (1.7)	1.6 (1.9)	<0.001#
ASA, *n* (%)			
1 and 2	391 (62.5)	452 (53.7)	<0.001+
3 and higher	235 (37.5)	389 (46.2)	<0.001 +
30 day mortality, n(%)	55 (8.8)	70 (8.3)	n.s.#

GC = geriatric comanagement

*n* = number

SD = standard deviation

CCI = Charlson comorbidity index

ASA = American Society of Anesthesiologists

^#^ = Students t-test

^+^ = Pearson chi-square test, Fisher exact test

### Patient requirements and further care after discharge

The total disposition in a nursing home was significantly higher in Group post-GC (3.9% vs. 1.7%, *p < 0*.*001*.). After discharge, less patients required a rehabilitation clinic in the post-GC group compared with the pre-GC group (8.2% vs. 12.0%, *p* < 0.001). Significantly more patients in the post-GC returned home after discharge (33.1% vs. 24.9%, *p* < 0.001) (Table 5).

**Table 5:** Disposition of patients after discharge.

	pre-GC	post-GC	*p-value*
*n*	626	841	
Returned home, *n* (%)	156 (24.9%)	278 (33.1%)	<0.001 +
Returned to same residence as before hospitalization, n (%)	375 (59.9)	414 (49.0)	0.032+
Rehabilitation unit, *n* (%)	75 (12.0%)	69 (8.2%)	<0.001+
Nursing home**, *n* (%)	11 (1.8%)	33 (3.9%)	<0.001 +
Retirement home**, *n* (%)	9 (1.4%)	47 (5.6%)	<0.001 +

GC = geriatric comanagement

*n* = number

** = Discharge to different residence as before hospitalization indicating the necessity of more intensive medical care

^+^ = Pearson chi-square test, Fisher exact test

### Outcome comparison according injury distribution

The sub-group analysis according to injury distribution revealed an increase in the number of patients, independent from injury distribution, and a decrease of LOS in geriatric patients with head injury (8.7 ± 3.9 days in Group pre-GC vs. 5.2 ± 4.9 days in Group post-GC, p = 0.041). Further, the mean CCI and the mean age-adjusted CCI were significantly higher in Group post-GC compared with Group pre-GC independent from injury distribution. More patient returned home after discharge in Group post-GC compared with Group pre-GC, independent of injury distribution (*p* < 0.05). The implementation of GC led to a decrease of the requirement of rehabilitation units after discharge in patients with extremity injuries (pre-GC 19.1% vs. post-GC 9.4%, *p* = 0.034) (Table 6).

**Table 6:** Comparison of clinical course and location after discharge stratified according to anatomic injury.

	Head			Trunk			Extremity		
	pre-GC	post-GC	*p-value*	pre-GC	post-GC	*p-value*	pre-GC	post-GC	*p-value*
	207	347		76	109		136	201	
Age [years], mean (SD)	80.7 (6.9)	82.2 (7.2)	0.014	80.5 (6.7)	84.7 (4.5)	0.02	80.9 (6.2)	85.5 (7.6)	0.046
LOS [day], mean (SD)	8.7 (3.9)	5.2 (4.9)	0.041	11.2 (10.5)	8.02 (6.0)	0.043	9.9 (12.3)	9.7 (6.5)	n.s.
Days from admission to definitive surgery, mean (SD)	2.2 (1.8)	3.2 (2.2)	n.s.	4.6 (4.5)	5.7 (10.7)	n.s.	1.5 (2.6)	1.8 (4.2)	n.s.
Days from first surgery to discharge, mean (SD)	8.1 (3.4)	9.5 (8.6)	n.s.	9.0 (6.8)	5.2 (8.7)	0.038	11.0 (3.8)	8.4 (6.5)	n.s.
CCI [points], mean (SD)	4.5 (1.5)	4.9 (1.7)	0.004	4.9 (2.1)	5.7 (2.3)	0.0467	4.6 (1.7)	5.2 (2.2)	0.01
CCI adjusted for age [points], mean (SD)	0.9 (1.4)	1.3 (1.5)	0.009	1.4 (1.9)	2.1 (1.1)	0.035	0.9 (1.6)	1.7 (2.1)	0.001
Nursing home*, n (%)	32 (15.5)	42 (12.1)	n.s.	14 (18.4)	8 (7.3)	0.036	4 (2.9)	16 (7.9)	0.036
Retirement home*, n (%)	47 (22.7)	63 (18.2)	0.043	18 (23.7)	29 (26.6)	n.s.	8 (5.9)	7 (3.5)	n.s.
Rehabilitation unit, n (%)	12 (5.8)	18 (5.2)	n.s.	15 (19.7)	15 (13.8)	n.s.	26 (19.1)	19 (9.4)	0.034
Returned home, n (%)	93 (44.9)	191 (55.0)	0.023	23 (30.3)	49 (44.9)	0.0436	76 (55.9)	129 (64.2)	0.017
Mortality, n (%)	23 (11.1)	33 (9.5)	n.s.	6 (7.9)	8 (7.3)	n.s.	8 (5.9)	7 (3.4)	n.s.

GC = geriatric comanagement

*n* = number

SD = standard deviation

LOS = length of stay

CCI = Charlson comorbidity index

n.s. = not significant

## Discussion

Geriatric trauma patients represent a challenge for orthopedic-trauma surgeons, as they may have multiple comorbidities, issues that can lead to complications (diabetes induced wound complications) and other alterations of the physiological response to trauma (osteoporosis induced cut out of implants) [31]. The interdisciplinary collaboration between trauma surgeons and geriatricians is thought to reduce the risk of complications. The aim of this study was to investigate the impact of demographic changes and the implementation of GC on geriatric trauma patient. Our results were as follows:

1. The number of geriatric trauma admissions increased by 34.3%, exceeding the demographic development (28.5%).
2. The mean number of non-trauma-related new diagnosis was three times higher per patient in the post-GC group.
3. Despite increased medical complexity, LOS decreased and the mortality rate remained comparable, with more patients able to return back home after discharge.
4. The implementation of GC increased the rate of patients returning back home while decreasing the requirement of rehabilitation units after discharge

Regarding our first result, our observed increase of geriatric trauma admission (by 34.3% within 8 years) was comparable with Lowe et al. [32] (+ 35% over the age of 65 years in a 9-year comparison).

Our increase of geriatric trauma patients is higher than found in the demographic changes of the population. The proportion of patients over the age of 65 years has increased by 28.5% from 2010 to 2018 according to the data from the federal bureau of statistics of Switzerland [33] and life expectancy of the general population increases, (4.4 years by 2040) [34]. Furthermore, injuries are estimated to be among the top ten leading causes of years of life lost by 2040 [34]. The results of this present study concur with these developments.

Regarding our second finding, the increase number of new diagnoses per geriatric trauma patient might be explained by a raising incidence of comorbidities. It is evident that ageing represent one main risk factor for the development of diseases [35], Yet, new and improved diagnostic tools improve diagnoses of pathological finding [36]. Both, aging and improved diagnostic tools (including laboratory tests, screening tools, and clinical scoring systems [22, 37]) might be responsible for the increase number of pathological conditions in our study population.

Regarding our third finding, advantages of interdisciplinary collaboration have been shown in several studies [38, 39]. The development of GC has led to improved healthcare and quality of life of the ortho-geriatric trauma patient [15, 40, 41]. Similar to our results, it has been shown that the implementation of multidisciplinary care improves clinical outcome of elderly patients after hip fracture [19, 42]. Despite some literature indicating no advantages of ortho-geriatric interventions [43], the majority of published studies support GC by showing improved outcomes [40, 42, 44]. The advantages of interdisciplinary comanagement is mirrored by the comparable mortality despite increased medical complexity.

The advantages of the implementation of GC is also shown by an increase of patients that are able to return to their home. The decrease of LOS might have been helpful in preventing complications, thus enabling more patients to return home safely [45]. Improved diagnostic tools and higher specialized treatment strategies may play a role as well [15].

### Limitations

We are aware of certain limitations of this study. First, the present study was conducted at a level one trauma center, which may induce a selection bias. Also, local specifics of rescue conditions have to be reflected. Second, we did not perform a sample size calculation prior the analysis. However, we included the maximum number of patients based on inclusion criteria and completeness of data in order to minimize a type II error. Third, the increase of geriatric trauma admission (aged 70 years and over) was compared with the increase of general elderly population (aged 65 years and over) based on publicly available data. One might argue that these groups are not comparable, the discrepancy of population-based increase of elderly and raise of geriatric trauma admission might be underestimated, thus supporting our conclusion.

The increasing number of patients requiring a nursing home represents a specific socioeconomic challenge for society. For the individual patient, trauma may have a significant impact [46] because the return to their normal living environment may be challenging or impossible, during the process of safe discharge to a skilled nursing facility rather than home [47]. The increased demand in specialised nursing homes for geriatric patients with increasing incidence in medical comorbidities and chronic medical conditions is confronted by the limited capacity of nursing facilities [48, 49] and will cause an economic challenge for the society.

## Conclusion

Our data appear to support the value of developing a GC for the following reasons. Despite increasing medical complexity the LOS decreased, mortality rate remained unchanged and more patients returned to home after in house treatment. In future studies, a multi center approach and a standardized geriatric trauma registry may be helpful in supporting our findings.

## Supporting information

S1 File STROBE statement—Checklist of items that should be included in reports of *cohort studies*. (DOCX)

S1 Fig Numbers/Percentages of patients per CCI point stratified according to the treatment group. The number of patients with increased CCI points is higher in Group post-GC when compared with Group pre-GC. Along with the increase of CCI, the proportion of patients increases in Group post-GC.
(TIF)

## Abbreviations

ASA: American Society of Anesthesiologists
ATLS: advance trauma life support
BMI: body mass index
CCI: Charlson comorbidity index
CI: confidence interval
ED: emergency department
EMR: Electronic medical record
GC: geriatric comanagement
GCS: Glasgow coma scale
ICD: international classification of disease
ICU: intensive care unit
LOS: length of stay
SD: standard deviation
SOP: standard operating protocol
