# Expression of Concern: The effect of geriatric comanagement (GC) in geriatric trauma patients treated in a level 1 trauma setting: A comparison of data before and after the implementation of a certified geriatric trauma center

**DOI:** 10.1371/journal.pone.0348035

**Published:** 2026-04-29

**Authors:** The PLOS One Editors

After this article [1] was published, the underlying data were removed from the publicly accessible repository (https://datadryad.org/stash/share/GB5cKxey9T08FDMPgZeg3L4mfBe_BIf8BuUMthuWwjE) and are currently not available. In response to editorial follow-up, the corresponding author stated that the underlying data are still available, but did not make the minimal dataset available or provide updated information on how to access it.

As such, this article [1] does not comply with the PLOS Data Availability policy and the *PLOS One* Editors issue this Expression of Concern.
