# Supplementary figures and images for: The effect of geriatric comanagement (GC) in geriatric trauma patients treated in a level 1 trauma setting: A comparison of data before and after the implementation of a certified geriatric trauma center
Source: PLoS One. 2021 Jan 11;16(1):e0244554. doi: 10.1371/journal.pone.0244554 (PMC7799827; doi:10.1371/journal.pone.0244554)

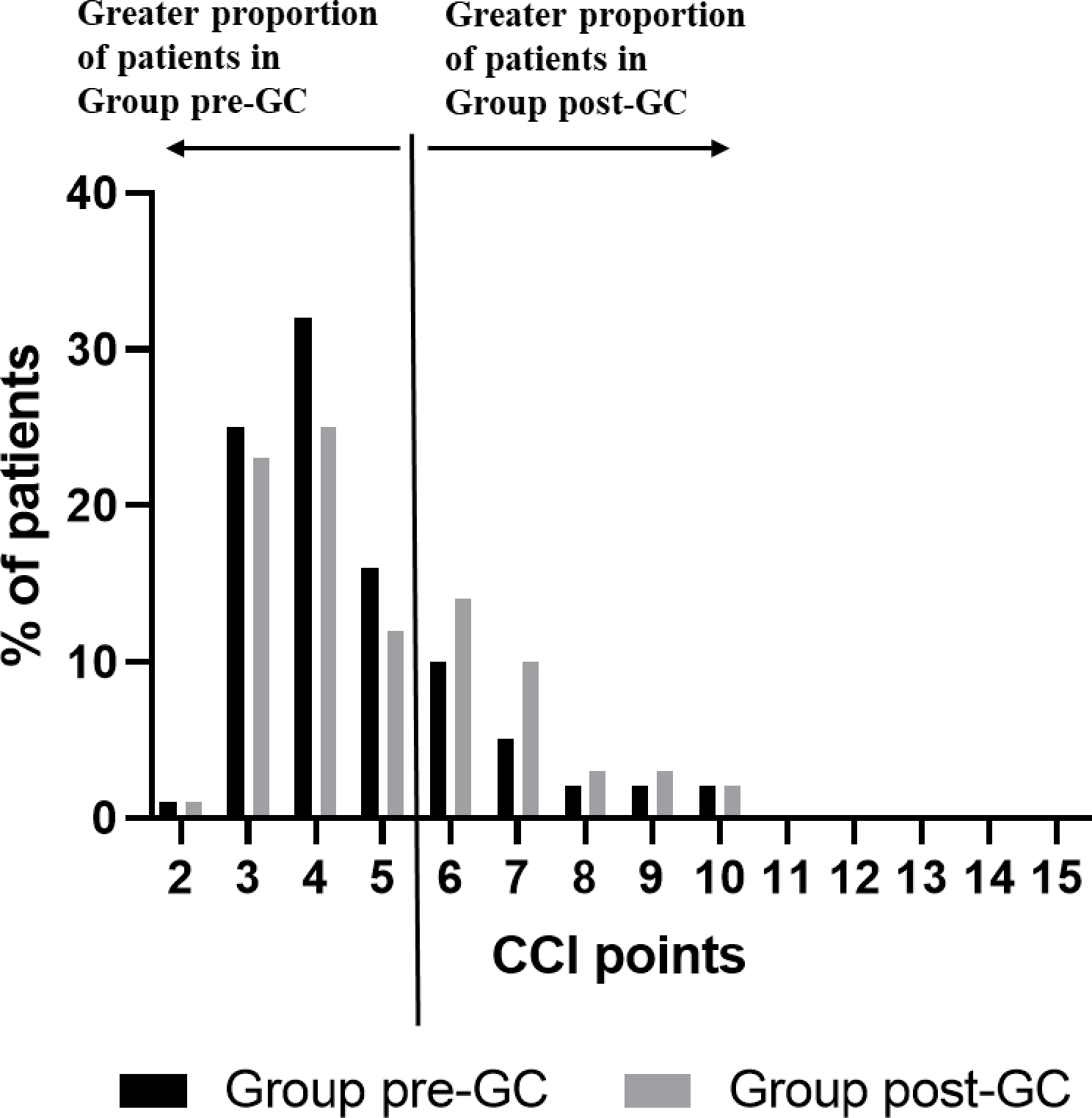

Supplement: S1 Fig — The number of patients with increased CCI points is higher in Group post-GC when compared with Group pre-GC. Along with the increase of CCI, the proportion of patients increases in Group post-GC. (TIF) [file pone.0244554.s002.tif]
